# The relationship between smokers' motivation to quit and intensity of tobacco control at the population level: a comparison of five European countries

**DOI:** 10.1186/1471-2458-8-2

**Published:** 2008-01-03

**Authors:** Jochen René Thyrian, Demosthenes B Panagiotakos, Evangelos Polychronopoulos, Robert West, Witold Zatonski, Ulrich John

**Affiliations:** 1 Institute of Epidemiology and Social Medicine, Ernst-Moritz-Arndt University of Greifswald, Walther-Rathenau-Str. 48, 17489 Greifswald, Germany; 2 Department Of Nutrition And Dietetics, Harokopio University, E Venizelou 70, 17571 Athens, Greece; 3 Department of Epidemiology and Public Health, University of London, Brook House 2-16 Torrington Place, London WC1E 6B, UK; 4 Cancer Epidemiological and Prevention Division, Cancer Center – Institute of Oncology, ul Roentgena 5, 02-781 Warsaw, Poland

## Abstract

**Background:**

Smoking prevalence differs significantly across Europe. In addition, there are considerable differences in tobacco control activities across European countries. The relationship between prevalence and policy is under-researched. The present analysis examines the motivation to change smoking behaviour across 5 different European countries that differ considerably in their tobacco control activities.

**Methods:**

A population-based, representative survey of 1750 smokers, aged 16–59, from 5 different European countries (Germany, Greece, Poland, Sweden, UK) was used. Demographic variables, smoking status and the motivation to stop smoking were assessed. Motivation was assessed as, first, intending to quit (using the stages of change plus a modified stage for Precontemplation), and second, the desire to quit.

**Results:**

The majority of smokers want to stop smoking (73.5%), while only 35.0% want to stop definitely. Across countries, 10.2% definitely do not want to stop.

Most of the smokers can be categorised in the Precontemplation stage (between 62.6% and 77.7% depending on the country), one of the stages of change categories. The relationship between the stages of change and the country under examination is statistically significant (chi-square = 43.466, p < 0.001). In countries with a high level of tobacco control, the proportion of people in Precontemplation is lower than in countries with low tobacco control activity.

**Conclusion:**

There are differences in the stages of change between the countries under examination. However, the categorisation of the countries into low, medium and high tobacco control activity used in this analysis does not explain these differences. Most smokers want to stop smoking, but a high proportion cannot indicate a time-frame when this is going to happen. Tobacco control efforts or other kinds of support might encourage these smokers to actually try to stop. Longitudinal studies at the population level are needed to assess, relate or monitor tobacco control activities and the intention to stop.

## Background

Smoking prevalence across Europe differs significantly [1] and there are considerable differences in tobacco control activities across countries as well [2,3]. The objective of tobacco control is to decrease the prevalence of smoking and consequently to reduce tobacco-attributable morbidity and mortality [4,5]. In pursuing this, tobacco control activities aim at supporting those who are motivated to stop their smoking behaviour. It has been argued that smoking prevalence is related to the overall motivation to change smoking with a low prevalence of smoking being associated with a more favourable stage distribution [6]. In other words, more people are motivated to quit. A higher prevalence of cigarette smoking is associated with a lower motivation to quit smoking, fewer quit attempts, and higher cigarette consumption among smokers [7]. Furthermore, hard-core smokers are coming to dominate the remaining population of smokers, [8] and thus they represent a challenge because they appear to be largely unaffected by the messages of tobacco control [9].

To our knowledge no studies exist that relate tobacco control and the motivation to stop smoking across different European countries, and therefore the objective of this work is to examine the motivation to stop smoking behaviour across 5 different European countries that differ considerably in their tobacco control.

## Methods

### Sample

This work is part of the project "European Survey on Tobacco Control Attitudes and Knowledge (ESTA)", a population-based, representative telephone survey of 3500 participants, aged 16–59, from five different European countries (Germany, Greece, Poland, Sweden, UK), and it was conducted by the Institut für Angewandte Sozialforschung GmbH (infas) between January and April 2006. The survey was created in English and then translated into each country's appropriate language. To assure validity of the translations, all five countries re-translated the survey into English before the final version was translated. Ethical approval for this survey was obtained from the funding organisation.

Regions were chosen to represent countries with low (Germany, Greece), medium (Poland) and high (Sweden, UK) tobacco control activity according to previous reports [2,3]. In the first report [2], a questionnaire was sent to correspondents in 30 European countries. The UK was ranked 2^nd^, Sweden 6^th^, Poland 12^th^, Greece 20^th ^and Germany 22^nd ^among 30 European countries using a scoring system designed with the help of a panel of international tobacco control experts,. The second report [3] is based on a questionnaire distributed at the World Conference of Tobacco or Health in Helsinki 2003 ranked Sweden 2^nd^, UK 4^th^, Greece 13^th ^and Germany 14^th ^of 14 member states of the EU. At that time, Poland was not a member of the EU. According to the WHO report for the European region, the most recent prevalence of adult smoking was 37.6% in Greece, 33.9% in Germany, 33.0% in Poland, 25.0% in the UK and 15.9% in Sweden [10]. However, prevalence data from the WHO-report differ in their assessment (year, age-group, smoking criteria) so that they are not easily comparable.

A household sample was randomly drawn from the national telephone book as well as a list of mobile phones. Using computer-assisted telephone interviews, target persons (age 16–59) were identified using the "last birthday method". Informed consent to participate was obtained via telephone. The sample was stratified for smoking status to compare smokers and non-smokers with equally sized groups. The response rate was 55% in Germany, 80% in Greece 72% in Poland, 79% in Sweden and 52% in the UK. For the present analysis, data is available from 350 smokers in each country.

Participants were asked for socio-demographic variables (age, sex, marital status) and smoking related characteristics (smoking status, urge to smoke and exposure to passive smoke, motivation to stop). The questionnaire used in this survey is available on the internet [11], sample characteristics are given in Table 1. The mean age (standard deviation) in the sample was 40.2 (11.89) years in Germany, 38.2 (10.99) in Greece, 40.3 (12.60) in Poland, 39.7 (11.99) in Sweden and 39.2 (11.98) in the UK; 56% of the participants were female (Germany 51%, Greece 58%, Poland 60%, Sweden 58%, UK 55%). In Germany, 64% of those polled lived in a relationship (49% in marriage), in Greece 68% (58%), in Poland 67% (60%), in Sweden 63% (58%) and in the UK 58% (42%).

**Table 1:** Sample characteristics

		Germany	Greece	Poland	Sweden	UK
	n	700	700	700	700	700
Age (in years)	Mean	40.2	38.2	40.3	39.7	39.2
	SD	11.89	10.99	12.60	11.99	11.98
Sex	Female	51%	58%	60%	58%	55%
Marital status ^b^	Any (married)	64% (49%)	68 (58%)	67% (60%)	63% (58%)	58% (42%)
Smoking status ^a^	Daily smokers	26.0%	40.4%	28.0%	14.4%	15.0%
	Hard-core smokers	7.6%	24.6%	18.9%	16.0%	17.2%
	Occasional smokers	7.1%	8.4%	6.6%	5.7%	4.8%
	quitters	1.6%	0.9%	2.3%	0.6%	1.7%

^a ^Smoking status was assessed by asking "Have you smoked cigarettes in the last six months?" with the response pattern (a) at least one cigarette per day for daily smokers, (b) occasionally for occasional smokers and (c) no, no cigarettes for non-smokers. To further confirm the status we asked "And is this the status today?" with people indicating "no" being categorized as quitters. Participants were asked when they smoke their first cigarette in the morning and are considered hard-core smokers when this happens in the first 5 minutes after waking.

^b ^Marital status: any = living in any relationship; married = being married

According to this survey the prevalence of daily smokers in Germany is 26.0%, in Greece 40.4%, in Poland 28.0%, in Sweden 14.4% and in the UK 15.0%. Of these smokers 17.0% can be considered hard-core smokers (Germany 7.6%, Greece 24.6%, Poland 18.9%, Sweden 16.0% and UK 17.2%). The prevalence of occasional smoking is as follows: in Germany 7.1%, in Greece 8.4%, in Poland 6.6%, in Sweden 5.7% and in the UK 4.8%. At the time of the assessment the prevalence of quitters was: in Germany 1.6%, in Greece 0.9%, in Poland 2.3%, in Sweden 0.6% and in the UK 1.7%.

### Data assessment

Smoking status was assessed by asking "Have you smoked cigarettes in the last six months?" with the response option (a) at least one cigarette per day for daily smokers, (b) occasionally for occasional smokers and (c) no, no cigarettes for non-smokers. To further confirm the status, we asked "And is this the status today?" with people indicating "no" being categorized as quitters. Participants were asked when they smoke their first cigarette in the morning and are considered hard-core smokers when this happens in the first 5 minutes after waking [12,13].

The motivation to stop smoking was measured using the stage of change construct. This was measured using a modified stages of change algorithm by DiCelemente [14] with further differentiation of the precontemplation stage [15]. The stage of change is a key variable for the design of individual and public health interventions for smoking cessation. It is a variable that employs past behaviour and behavioural intention to characterize an individual's readiness to change. As a single-item survey measure, it has shown that it is readily applicable to population studies and appears to provide important information about the population characteristics of readiness to change behavioural risk factors [16]. The pattern of stage distribution has shown to be stable across different samples and ages. Differences were found with respect to education, Hispanic origin, race [17] and country [6]. A longitudinal analysis in the US has shown that the pattern was static across time and highlights that tobacco control efforts must receive high priority to address this [18].

Since there has been recent discussion about staging, and it has been stated to revert to simple questions about desire to change [19], we also asked if people wanted to stop smoking and made them indicate (a) yes, definitely, (b) yes, probably, (c) no, I would rather not, or (d) no, definitely not. Their answers provided a measure of their motivation to stop smoking.

### Statistical Analysis

Proportions and their 95%-confidence intervals were calculated using the "Confidence Interval Analysis Disk Version 2.0.0 [20]."

To test relations between countries and the stages of change, a 3 × 5 chi-square test was calculated for the 3 stages of change (PC, C, PR) and the 5 countries. Then we partitioned the table into four 3 × 2 tables comparing (a) Germany and Greece, (b) Sweden and the UK, (c) Germany and Greece with Poland and (d) Poland with the UK and Sweden.

The same procedure was applied to test for differences between countries in the subgroups of the PC stage, resulting in a 4 × 5 chi-square test and for the desire to quit smoking, resulting in another 4 × 5 chi-square test.

## Results

The stages of change categorise most of the smokers in the precontemplation stage (between 62.55% and 77.3% depending on the country). There are considerable differences between countries in the frequency of the preparation stage with 18.9% of the smokers of Poland indicating a desire to quit during the next 4 weeks, while this is only true for 8.9% of the smokers from Germany. Subdividing the precontemplation stage delivers a more diverse distribution of smokers. German smokers had the greatest proportion (21.1%) who indicated that they never intend to quit smoking. In Greece nearly half of the smokers indicated a desire to quit sometime in the future (46.7%). This category was the most prevalent across all countries with 31.6% of smokers being placed in this category (Table 2). There are no gender or age differences.

**Table 2:** The desire to stop smoking and the intention to quit smoking in 5 different European countries for n = 1750 smokers

Variable			Germany	Greece	Poland	Sweden	UK	Total
			%	%	%	%	%	%
Level of tobacco control activity			low	low	medium	high	high	
Stage of change								
When do you intend to quit								
	Preparation	during the next 4 weeks	8.9	14.1	18.9	9.4	14.8	13.2
	Contemplation	during the next 6 months	13.3	9.3	18.5	24.8	18.2	16.6
	Precontemplation		77.7	76.7	62.6	65.8	67.0	70.2
		*during the next 12 months*	*15.6*	*7.8*	*19.3*	*10.3*	*20.8*	*14.6*
		*during the next 5 years*	*14.5*	*8.2*	*6.6*	*10.3*	*8.9*	*9.7*
		*sometime in the future*	*26.6*	*46.7*	*26.8*	*31.2*	*25.4*	*31.6*
		*never*	*21.1*	*14.1*	*9.9*	*14.1*	*11.9*	*14.3*
Do you want to stop smoking?								
	yes, definitely		29.8	23.5	33.2	46.9	44.3	35.0
	yes, probably		34.5	42.5	44.5	35.8	34.4	38.5
	no, rather not		23.3	17.2	13.6	6.2	13.9	15.0
	no definitely not		11.1	16.1	6.4	10.7	6.1	10.2

The relationship between stages of change and countries is statistically significant with an overall chi-square of 43.466 (df = 8, p < 0.001). There is a significant difference between Germany and Greece (chi-square = 9.660, df = 2, p < 0.01), Sweden and the UK (chi-square = 24.002, df = 2, p < 0.001), countries with medium and high tobacco control profile (chi-square = 7.214, df = 2, p < 0.05). However, there is not a significant difference between countries with low and moderate tobacco control profile (chi-square = 5.702, df = 2, p > 0.05).

The relationship between the differentiation of the Precontemplation stage and countries yields statistically significant results with an overall chi-square of 61.084 (df = 12, p < 0.001.). There is a significant difference between Germany and Greece (chi-square = 14.456, df = 3, p < 0.01), Sweden and the UK (chi-square = 10.393, df = 3, p < 0.05), countries with low and moderate tobacco control profiles (chi-square = 32.106, df = 3, p < 0.001), and countries with medium and high tobacco control profiles (chi-square = 21.845, df = 3, p < 0.001).

The majority of smokers want to stop smoking (73.5%), but only 35.0% want to stop definitely. Across countries, an average of 10.2% of smokers definitely do not want to stop. In countries with comparably high tobacco controls (UK, Sweden) the percentage of smokers who definitely want to quit is higher than in countries with low tobacco control (Germany, Greece). By the numbers, 44.3% or 46.9% vs. 29.8% or 23.5%, respectively, want to quit. The fewest smokers who definitely do not want to stop (6.1% and 6.4%) are in the UK and Poland, respectively, while this percentage is much higher in Greece (16.1%). The detailed results are given in Table 2.

The relationship between the desire to quit smoking and countries is statistically significant with an overall chi-square of 78.684 (df = 12, p < .001.). There is a significant difference between Germany and Greece (chi-square = 13.943, df = 3, p < 0.01), Sweden and the UK (chi-square = 10.531, df = 3, p < 0.05), countries with low and moderate tobacco control profiles (chi-square = 13.567, df = 3, p < 0.01) and countries with medium and high tobacco control profiles (chi-square = 42.670, df = 3, p < 0.001).

## Discussion

There are significant differences across European countries in the stages of change for quitting smoking, with Germany and Greece showing a higher proportion of people in the Precontemplation stage than Greece, the UK and Sweden. A crude view of the countries categorised according to their tobacco control profiles suggests that in countries with a high level of tobacco control activity, the proportion of people who are intending to quit in a set time-frame of 6 or 12 months is higher than in countries with low tobacco control activity. However, Poland was initially identified as a country with a mid-level of tobacco control. It does not show a distribution of the stages of change that is somewhere in between the high and low level countries, thus this explanation might be too short-sighted. On the one hand, the definition of level of tobacco control policy used here might be misleading and not well chosen for the research question. In particular, the comparisons between the stages of change in the UK and Sweden (high level) became statistically significant as well as the comparison between the stages of change between Greece and Germany (low level). On the other hand, other factors not examined here might be important as well. One possible explanation might be cultural differences in response patterns or differences in the social desirability of smoking.

This explanation might be supported by the results when the Precontemplation stage is further differentiated into stages determined by the definite time frame in which people intend to quit. The differences between countries and also levels of tobacco control policy are statistically significant. In general, a third of the smokers indicate intending to quit sometime, leaving much room for speculation as to whether their wish to quit or their effort to quit is really genuine. On the other hand, this might indicate that these smokers are waiting for the right time, the right occasion or the right support. This result is in line with current research that stopping smoking is not clearly formulated by people or occurs in an unplanned manner. However, there are cultural differences in response patterns across European Countries. For example, in Greece nearly half of the smokers do not commit to a definite time or to a definite "never," while this proportion is much lower in Poland. The question of whether setting a time-frame in the operationalisation of the stages of change is culturally fair cannot be answered by this analysis; however, further research needs to be conducted that enables an interpretation of these differences.

The majority of smokers in Germany, Greece, Poland, Sweden and the UK want to stop smoking. This is a promising result for tobacco control efforts in the participating countries because it implies that the population might welcome efforts that will help them to stop smoking. It also implies that campaigns about the consequences of smoking have been fruitful since the awareness of smoking as an unhealthy/unwanted behaviour seems to be present. The proportion of smokers who indicate definitely not wanting to stop is rather small. This might be a group of smokers that is unreachable by tobacco control measures, or at least disagrees with many measures that cut their opportunities to smoke. However, realising that this is just about every 10^th ^smoker supplies an optimistic outlook for tobacco control advocates.

## Limitations

There are limitations to this analysis. First, the concept of the stages of change has been debated, its measurement has been called arbitrary and there has been a call for abandoning the underlying model [19]. We used a modified stage algorithm that was found to be predictive of quitting activity after 7 months, depending on different quitting plans [15]. We also added a simple assessment of the motivation to change smoking to further differentiate motivation into wanting and intending to quit. Second, the common limitations for telephone surveys of smoking variables apply to this study (retention, missing biochemical validation, validity, etc.). However, conducting assessments across five different countries by phone is the most cost-effective way to gather this information and constitutes a state of the art approach. There might also be a selection bias, since females are over represented, even though we chose the last birthday method. Third, tobacco control activities may take years to have an effect, so one needs to look not just cross-sectionally, but historically as well as concurrently. Fourth, our definition of tobacco control activity and therefore the selection of the countries for the survey might not be well chosen for the research question as previously discussed. A research design evaluating a greater variety of countries and other criteria for selection might be needed.

## Conclusion

The differentiation into countries with low, medium and high tobacco control activity chosen in this analysis does not explain differences in the motivation to change smoking habits. However, there are differences between countries in motivation to quit smoking and in all countries there are a small but substantial proportion of current smokers who do not want to stop smoking. This remains a challenge for tobacco control.

However, most smokers want to quit smoking, even though a high proportion cannot indicate a time-frame for when this is going to happen. Tobacco control efforts or other kinds of support might encourage these smokers to actually try to quit. Longitudinal studies are needed that relate or monitor tobacco control activities and the intention to change on a population level.

## Competing interests

The author(s) declare that they have no competing interests.

## Authors' contributions

JT conceived the study, performed the statistical analyses and drafted the manuscript. DP participated in the statistical analyses. EP participated in the design of the study and was responsible for the Greek survey. RW participated in the design of the study, contributed to the theoretical framework and was responsible for the survey in the UK. WZ participated in the design of the study and was responsible for the Polish survey. UJ coordinated the study in general and was responsible for the German part of the study. All authors read and approved the final manuscript.

## Pre-publication history

The pre-publication history for this paper can be accessed here:



## References

[B1] Shafey O, Dolwick S, Guindon GE (2003). Tobacco Control Country Profiles 2003.

[B2] Joossens L, Raw M (2006). The Tobacco Control Scale: a new scale to measure country activity. Tob Control.

[B3] Thyrian JR, John U (2006). Measuring activities in tobacco control across the EU. The MAToC. Subst Abuse Treat Prev Policy.

[B4] Frieden TR, Mostashari F, Kerker BD, Miller N, Hajat A, Frankel M (2005). Adult tobacco use levels after intensive tobacco control measures: New York City, 2002-2003. Am J Public Health.

[B5] Biener L, Harris JE, Hamilton W (2000). Impact of the Massachusetts tobacco control programme: population based trend analysis. Bmj.

[B6] Etter JF, Perneger TV, Ronchi A (1997). Distributions of smokers by stage: international comparison and association with smoking prevalence. Prev Med.

[B7] Etter JF (2004). Associations between smoking prevalence, stages of change, cigarette consumption, and quit attempts across the United States. Prev Med.

[B8] Warner KE, Burns DM (2003). Hardening and the hard-core smoker: concepts, evidence, and implications. Nicotine Tob Res.

[B9] Emery S, Gilpin EA, Ake C, Farkas AJ, Pierce JP (2000). Characterizing and identifying "hard-core" smokers: implications for further reducing smoking prevalence. Am J Public Health.

[B10] WHO regional office for Europe European health for all database (HFA-DB) - Updated: June 2007. http://www.who.dk/document/tob/tobconf2002/edoc8.pdf.

[B11] Institute of Epidemiology and Social Medicine (2006). EU-Project - Measuring Tobacco Control from the General Population Perspective - European Survey On Tobacco Control Attitudes And Knowledge Introduction – ESTA. http://www.medizin.uni-greifswald.de/epidem/engl/eu/esta/index.htm.

[B12] Heatherton TF, Kozlowski LT, Frecker RC, Fagerstrom KO (1991). The Fagerstrom Test for Nicotine Dependence: a revision of the Fagerstrom Tolerance Questionnaire. Br J Addict.

[B13] John U, Meyer C, Schumann A, Hapke U, Rumpf HJ, Adam C, Alte D, Ludemann J (2004). A short form of the Fagerstrom Test for Nicotine Dependence and the Heaviness of Smoking Index in two adult population samples. Addict Behav.

[B14] Prochaska JO, DiClemente CC (1992). Stages of change in the modification of problem behaviors. Prog Behav Modif.

[B15] Dijkstra A, De Vries H (2000). Subtypes of precontemplating smokers defined by different long-term plans to change their smoking behavior. Health Educ Res.

[B16] Laforge RG, Velicer WF, Richmond RL, Owen N (1999). Stage distributions for five health behaviors in the United States and Australia. Prev Med.

[B17] Velicer WF, Fava JL, Prochaska JO, Abrams DB, Emmons KM, Pierce JP (1995). Distribution of smokers by stage in three representative samples. Prev Med.

[B18] Wewers ME, Stillman FA, Hartman AM, Shopland DR (2003). Distribution of daily smokers by stage of change: Current Population Survey results. Prev Med.

[B19] West R (2005). Time for a change: putting the Transtheoretical (Stages of Change) Model to rest. Addiction.

[B20] Altman DG, Machin D, Bryant TN, Gardner MJ (2000). Statistics with Confidence - 2nd edition.

